# Longitudinal Dynamics of Physical Function With Anxiety and Depression in Parkinson's Disease: A Cross‐Lagged Panel Analysis of the PPMI Dataset

**DOI:** 10.1002/brb3.71257

**Published:** 2026-02-12

**Authors:** Philip Hodgson, Alastair Jordan, Charikleia Sinani, Divine Charura, Adam Hodgson, Hanna L. Glandorf

**Affiliations:** ^1^ Department of Physiotherapy Tees, Esk and Wear Valleys NHS Foundation Trust, West Park Hospital Darlington UK; ^2^ School of Science, Technology and Health York St John University York UK; ^3^ School of Education, Language and Psychology York St John University York UK; ^4^ Independent Researcher; ^5^ Lohfert & Lohfert Hamburg Germany

**Keywords:** mental health, Parkinson's disease, physical health, physiotherapy, symptom interaction

## Abstract

**Introduction:**

Parkinson's disease (PD) is characterized by a complex interplay of motor and non‐motor symptoms. While cross‐sectional studies have shown a link between psychological symptoms and self‐reported physical function, the longitudinal and directional nature of this relationship remains unclear. This study aimed to clarify the temporal relationships between psychological symptoms (depression and anxiety) and physical function (both participant‐reported and clinician‐rated) in individuals with PD.

**Methods:**

We used a rigorous longitudinal analytical approach, random‐intercept cross‐lagged panel modeling (RI‐CLPM), on data from 1128 individuals with PD from the Parkinson's Progression Markers Initiative (PPMI) dataset. We examined directional cross‐lagged paths between psychological symptoms (via GDS and STAI) and physical function measures (via MDS‐UPDRS Parts II and III).

**Results:**

Our analysis revealed significant bidirectional cross‐lagged paths between depressive symptoms and participant‐reported physical function. A worsening of depression scores predicted a subsequent decline in participant‐reported physical function (via MDS‐UPDRS Part II), and vice versa. In contrast, no significant cross‐lagged paths were found between psychological symptoms and clinician‐rated motor function (via MDS‐UPDRS Part III). The analysis also showed a significant unidirectional path from participant‐reported function to future clinician‐rated function, suggesting that a participant's self‐perception of disability may precede objective motor decline.

**Conclusions:**

Our findings reveal a bidirectional relationship between depressive symptoms and an individual's own reporting of their physical function. This emphasizes the important role of patient‐reported outcomes as an indicator of PD progression. Therefore, we advocate for an integrated, multidisciplinary approach in clinical practice, where mental health screening and support are included in standard PD care.

## Graphical abstract

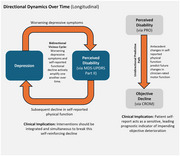

## 1 Introduction

Parkinson's disease (PD) is a progressive neurodegenerative disorder characterized by motor and non‐motor symptoms, including bradykinesia, tremor, rigidity, anxiety, and depression (Hodgson et al. 2025a; Prasad and Hung 2021; Bloem et al. 2021). In addition to its hallmark motor impairments, PD frequently involves psychological symptoms that can substantially impact quality of life and functional independence (Candel‐Parra et al. 2021; Gökçal et al. 2017).

Non‐motor symptoms like anxiety and depression are prevalent in PD and affect over 40% of individuals (Reijnders et al. 2008). These psychological factors are not merely secondary concerns, but lead to tangible, negative consequences such as social withdrawal (Ahn et al. 2022), reduced adherence to treatment regimens (Radojević et al. 2022), and increased emotional and practical strain on family members and caregivers (Goldstein et al. 2023). Therefore, a deeper understanding of the interplay between physical and psychological symptoms is essential for providing truly holistic, patient‐centered care.

Recent cross‐sectional analyses (Hodgson et al. 2025a) showed that depression and anxiety are associated with worse self‐reported physical function in people with PD (PwP). Notably, these relationships were considerably stronger for patient‐reported motor impairment than for clinician‐rated motor impairment, highlighting the importance of patient perspectives in the assessment of functional health. Discrepancies between patient and clinician‐rated outcomes suggest that psychological factors may influence how PwP perceive and report their physical abilities, raising questions regarding directionality and causality.

While a clinician may observe and measure a patient's physical abilities, psychological factors like anxiety and depression may influence a patient's own perception of their physical functioning (Hodgson et al. 2025a). This highlights the crucial role that a patient's subjective experience plays in their overall health and well‐being. Such discrepancies underscore the need for patient‐reported outcomes (PROs) to be given more weight in clinical practice and research to develop more effective, individualized treatment strategies.

Single‐time‐point (cross‐sectional) evaluations are inherently limited in their capacity to reveal how symptoms influence each other as conditions progress (Fischer et al. 2013). Clarifying the longitudinal relationships between psychological symptoms and physical functioning could improve the timing, targeting, and evaluation of both medical and psychosocial interventions in PD (Hodgson et al. 2024a; Hodgson et al. 2024b Hodgson et al. 2025a; Hodgson et al. 2025b; Hodgson et al. 2025c). A better appreciation of these associations is necessary as guidelines increasingly call for multidisciplinary, individualized strategies addressing both motor and mental health needs (Bloem et al. 2020).

Longitudinal analytical approaches, such as cross‐lagged panel modeling (CLPM), offer the opportunity to study disease progression over time, revealing dynamic, potentially bidirectional influences between psychological symptoms and physical function (Usami et al. 2019). The inclusion of random intercepts is a key feature of the RI‐CLPM that distinguishes it from a traditional CLPM. A standard CLPM can sometimes lead to a misleading result, where a significant cross‐lagged effect is found when the true relationship is simply a stable, time‐invariant covariance between variables at the between‐person level. The RI‐CLPM addresses this by modeling and separating these between‐person effects from the true within‐person, temporal effects (Hamaker et al. 2015).

The appreciation of the complex relationship between physical and psychological symptoms is particularly timely because modern treatment and psychotherapeutic intervention guidelines increasingly advocate for a more holistic, patient‐centered approach to care (National Institute for Health and Care Excellence 2024; AAN. Parkinson 2020). This trend moves away from a sole focus on motor symptoms and recognizes the profound impact of non‐motor symptoms on a patient's overall well‐being. For example, recent guidelines from organizations like the National Institute for Health and Care Excellence (NICE) (2024) and the American Academy of Neurology (AAN) (2020) call for the integration of mental health screening into the standard care pathway for PD patients. By exploring the temporal dynamics of these symptoms, our research directly supports this paradigm shift, providing evidence that can inform the development of more effective, personalized, and multidisciplinary strategies to improve clinical outcomes and quality of life for individuals with PD.

This study extends previous findings by employing CLPM analysis on repeated assessments from the Parkinson's Progression Markers Initiative (PPMI) cohort (Marek et al. 2018). This dataset is well‐suited for our analysis due to its extensive and longitudinal nature, providing a variety of information on a large and diverse group of individuals with PD. The PPMI's comprehensive data collection, which includes both motor and a wide range of non‐motor symptoms like anxiety and depression, allows us to conduct a detailed and robust investigation into their dynamic relationship. Using this cohort strengthens the generalizability of our findings.

Our aim is to explore the temporal inter‐relationships between physical function (as rated by both participants and clinicians) and psychological symptoms (anxiety and depression) over time in PD. We seek to clarify the direction and magnitude of these associations, providing new insights into how psychological and motor symptoms co‐evolve as PD progresses, informing both future research and clinical care strategies.

## 2 Methods

### 2.1 Ethical Approval

Ethical approval was obtained via York St John University (ETH2324‐0171). Informed patient consent was not necessary for this work.

### 2.2 Study Design, Data Source, and Selection

This study conducted a multi‐timepoint longitudinal analysis using data from the PPMI, a large, international, multi‐center observational study designed to identify biomarkers and track the clinical progression of PD. For further details, see Hodgson et al. (2025a) and www.ppmi‐info.org. The dataset included repeated measurements for a wide range of motor and non‐motor features in individuals living with PD, as well as demographic and clinical characteristics.

Individuals were included if they had a clinical diagnosis of PD as per the PPMI dataset (December 2023 data cut). For this analysis, participants were required to have data at two or more timepoints to facilitate longitudinal modeling. For RI‐CLPM analysis, data were restricted to the first four data collection timepoints: Baseline, V04 (12 months), V06 (24 months), and V08 (36 months).

### 2.3 Measures

Outcomes of interest included a range of reliable and valid physical and psychological outcomes used in PD (Hoehn and Yahr 1967; Nasreddine et al. 2005; Spielberger et al. 1971; Yesavage and Sheikh 1986; Goetz et al. 2008), alongside additional outcomes including age, sex, disease duration, and Montreal Cognitive Assessment (MoCA). These outcomes are summarized in Table 1. The approach to measurement and operationalization of these outcomes aligns with previous cross‐sectional work (Hodgson et al. 2025a).

**TABLE 1:** Outcomes of interest.

Physical function	Psychological symptoms	Other
Participant‐reported: MDS‐UPDRS Part II (self‐report of motor experiences of daily living) (Goetz et al. 2008)	Depressive symptoms: 15‐item geriatric depression scale (GDS) (Yesavage and Sheikh 1986)	Montreal cognitive assessment (MoCA) (Nasreddine et al. 2005)
Clinician‐rated: MDS‐UPDRS Part III (clinician motor examination) (Goetz et al. 2008)	Anxiety: State‐trait anxiety inventory (STAI) (Spielberger et al. 1971)	Disease duration (years since diagnosis)
Hoehn & Yahr (H&Y) stage (Hoehn and Yahr 1967)		Age
		Sex

### 2.4 Data Processing and Statistical Analysis

Data were extracted from the PPMI database and checked for completeness and plausibility. All statistical analyses were carried out using R and R Studio (Version 2024.120 + 467) and checked using Python (Version 3.13). Descriptive statistics were generated for all included variables at each timepoint. Missing data were considered on a pairwise basis, with no imputation. The amount of missing data varied between each variable and is reported within the results section. The models were then run, using a Yuan–Bentler estimator to assist with managing missingness. Demographic summaries of variables and correlations between variables across timepoints were also completed.

### 2.5 Random‐Intercept Cross‐Lagged Panel Modeling (RI‐CLPM)

The primary analysis employed random‐intercept cross‐lagged panel modeling (RI‐CLPM) to examine the temporal and directional relationships between 15 different variable combinations. The combinations of interest were chosen to consider the effect of overall PD stage, physical function (individual‐ and clinician‐assessed), depression, anxiety, and cognition on one another. In line with the Yuan‐Bentler method (Yuan and Bentler 2000), an estimator was used to help consider the impact of missing data and provide an indication of the model's fit. This approach allows for the simultaneous examination of within‐person changes over time and stable between‐person differences.

For each of the variable combinations, we first fitted a standard, unconstrained RI‐CLPM. This model included random intercepts that were permitted to co‐vary, which accounts for the constant association between variables and isolates the effects of time‐invariant confounders. The within‐person factor included autoregressions (stability over time), within‐time covariances (concurrent association), and cross‐lags (longitudinal directional influence) (Hamaker et al. 2015).

We also fitted a constrained RI‐CLPM for each combination based on the recommendations of Mulder and Hamaker (Mulder and Hamaker 2021). In this model, the autoregressive and cross‐lagged paths were fixed to be equal across all timepoints. This constraint was based on the theoretical assumption that the stability of each variable and the directional relationships between them would remain consistent over the study period.

To evaluate model fit, we used a combination of absolute and incremental fit indices, as recommended by Byrne (2001). The absolute indices were the root mean square error of approximation (RMSEA) and the standardized root mean square Residual (SRMR), while the incremental indices were the Tucker–Lewis Index (TLI) and the Comparative Fit Index (CFI). Acceptable and excellent fit thresholds were established based on similar prior research (Moen et al. 2019). Finally, we used χ^2^ difference tests to compare the fit of the standard and constrained models (Stoel et al. 2006). Effect sizes for the cross‐lagged paths were interpreted according to Orth et al. (Orth et al. 2024).

## 3 Results

**FIGURE 1:**
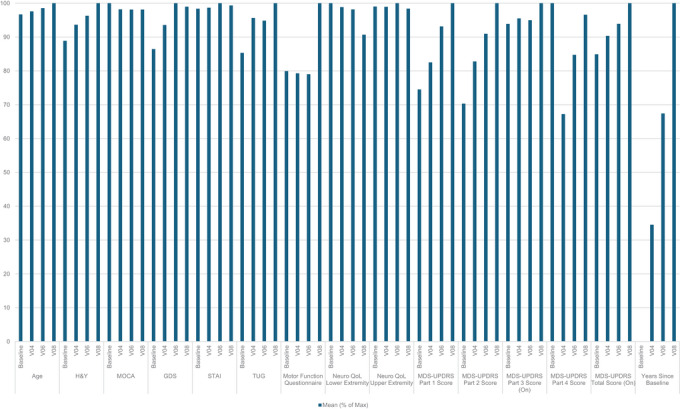
Normalized change in outcomes over time.

In the analysis, 1128 individuals with PD were included. Participant demographics and key outcomes at baseline and subsequent timepoints are summarized in Tables 2 and 3. Figure 1 displays the change in selected variables over time, normalized with respect to the maximum value.

**TABLE 2:** Participant demographics.

	Baseline			V04			V06			V08		
	*n*	Mean	SD	n	Mean	SD	n	Mean	SD	n	Mean	SD
**Age (years)**	1128	62.71	9.84	806	63.29	9.81	605	63.91	9.86	552	65.55	9.88
**Time since symptom onset (years)**	1113	2.98	3.18	793	3.98	2.78	594	5.14	3.65	541	5.96	3.33
**Time since diagnosis (years)**	1128	1.39	1.77	806	2.49	1.81	605	3.50	1.83	552	4.46	1.83
	Category	Count ( *n* )	Percentage (%)	Category	Count ( *n* )	Percentage (%)	Category	Count ( *n* )	Percentage (%)	Category	Count ( *n* )	Percentage (%)
**Sex^a^**	Female	441	39.10%	Female			Female			Female		
	Male	687	60.90%	Male			Male			Male		
**Race^a^**	White	1049	93.00%	White			White			White		
	Black	17	1.51%	Black			Black			Black		
	Asian	14	1.24%	Asian			Asian			Asian		
	Other	40	3.55%	Other			Other			Other		
	Unknown	8	0.71%	Unknown			Unknown			Unknown		
**Family Hx PD^a^**	Yes	399	35.37%	Yes			Yes			Yes		
	No	729	64.63%	No			No			No		
	Unknown	0	0.00%	Unknown			Unknown			Unknown		
**Handedness^a^**	Right	995	88.29%	Right			Right			Right		
	Left	102	9.05%	Left			Left			Left		
	Mixed	30	2.66%	Mixed			Mixed			Mixed		
	Unknown	0	0.00%	Unknown			Unknown			Unknown		

^a^Reported at baseline only.

**TABLE 3:** Outcome summary.

		*n*	Mean	SD
H&Y	Baseline	1115	1.68	0.51
	V04	776	1.77	0.55
	V06	574	1.82	0.56
	V08	520	1.89	0.58
MOCA	Baseline	1121	26.67	2.72
	V04	789	26.19	3.33
	V06	592	26.17	3.48
	V08	529	26.17	3.40
GDS	Baseline	1117	2.55	2.82
	V04	801	2.76	3.06
	V06	600	2.95	3.07
	V08	546	2.92	3.05
STAI	Baseline	1117	65.59	19.09
	V04	802	65.79	19.23
	V06	601	66.67	19.02
	V08	543	66.24	18.86
TUG	Baseline	16	10.76	3.15
	V04	13	12.06	2.20
	V06	16	11.96	2.08
	V08	33	12.61	3.04
Motor function questionnaire	Baseline	411	6.06	2.89
	V04	293	6.01	2.96
	V06	130	5.99	2.67
	V08	55	7.58	3.26
Neuro QoL lower extremity	Baseline	410	38.37	2.59
	V04	294	37.93	3.45
	V06	130	37.67	3.70
	V08	54	34.80	5.67
Neuro QoL upper extremity	Baseline	410	38.22	3.01
	V04	294	38.19	2.53
	V06	130	38.60	2.59
	V08	54	37.98	3.06
MDS‐UPDRS Part 1 score	Baseline	1117	6.52	4.87
	V04	801	7.22	4.97
	V06	604	8.15	5.34
	V08	551	8.75	5.63
MDS‐UPDRS Part 2 score	Baseline	1124	6.54	4.87
	V04	803	7.70	5.48
	V06	602	8.46	6.00
	V08	551	9.30	6.25
MDS‐UPDRS Part 3 score (On)	Baseline	1113	21.54	10.14
	V04	771	21.91	10.80
	V06	568	21.79	11.20
	V08	507	22.94	12.37
MDS‐UPDRS Part 4 score	Baseline	260	1.77	2.79
	V04	580	1.19	2.32
	V06	526	1.50	2.60
	V08	512	1.71	2.67
MDS‐UPDRS total score (On)	Baseline	1101	34.59	15.25
	V04	769	36.81	16.28
	V06	565	38.26	17.24
	V08	506	40.74	19.42
Years since baseline	Baseline	1128	0.00	0.00
	V04	806	1.05	0.12
	V06	605	2.05	0.12
	V08	552	3.04	0.11

Abbreviations: GDS = Geriatric Depression Scale; H&Y = Hoehn and Yahr Scale; MDS‐UPDRS = Movement Disorder Society‐Unified Parkinson's Disease Rating Scale; QoL = Quality of Life; STAI = State Trait Anxiety Inventory; TUG = Timed Up and Go Test.

### 3.1 Correlation Coefficients

The correlation matrix and filterable table, including the number of individuals whose data were used in calculating the respective coefficients, are provided in Supporting Information Files S1 and S2.

### 3.2 RI‐CLPM

For each variable combination, both an unconstrained (basic) and a constrained RI‐CLPM were fitted. The model fit indices (i.e., Robust CFI, Robust TLI, Robust RMSEA, and SRMR) were evaluated against established thresholds for fit. The indices of all model and variable combinations are provided in Table S1. Table S2 provides a direct comparison of the respective fits of each model for the specific variable combinations. Table 4 presents data for cross‐lags and covariances within the constrained models for each variable combination.

**TABLE 4:** Overview of estimated RI‐CLPMs.

	Constrained model *(BL, V04, V06, V08)*								
	Cross lags					Covariances (RIx∼∼RIy)			
Variable combination (X x Y)	Path	Contribution estimate	95% CI	*p* Value	Path		Contribution estimate	95% CI	*p* Value
H&Y x MOCA	wx2∼wy1	−0.030	−0.047 to −0.013	**0.001**	RIx∼∼RIy		−0.256	−0.434 to 0.077	**0.005 **
	wy2∼wx1	−0.485	−0.799 to −0.171	**0.002**					
	wx3∼wy2	−0.030	−0.047 to −0.013	**0.001**					
	wy3∼wx2	−0.485	−0.799 to −0.171	**0.002**					
	wx4∼wy3	−0.030	−0.047 to −0.013	**0.001**					
	wy4∼wx3	−0.485	−0.799 to −0.171	**0.002**					
H&Y x GDS	wx2∼wy1	0.014	−0.012 to 0.040	0.286	RIx∼∼RIy		0.146	0.032 to 0.259	**0.012 **
	wy2∼wx1	0.238	−0.235 to 0.711	0.324					
	wx3∼wy2	0.014	−0.012 to 0.040	0.286					
	wy3∼wx2	0.238	−0.235 to 0.711	0.324					
	wx4∼wy3	0.014	−0.012 to 0.040	0.286					
	wy4∼wx3	0.238	−0.235 to 0.711	0.324					
H&Y x STAI	wx2∼wy1	0.001	−0.002 to 0.005	0.420	RIx∼∼RIy		0.764	0.098 to 1.430	**0.024 **
	wy2∼wx1	0.375	−1.889 to 2.639	0.745					
	wx3∼wy2	0.001	−0.002 to 0.005	0.420					
	wy3∼wx2	0.375	−1.889 to 2.639	0.745					
	wx4∼wy3	0.001	−0.002 to 0.005	0.420					
	wy4∼wx3	0.375	−1.889 to 2.639	0.745					
H&Y x UPDRS II	wx2∼wy1	0.027	0.017 to 0.038	**<0.001 **	RIx∼∼RIy		0.563	0.317 to 0.808	**<0.001 **
	wy2∼wx1	0.470	−0.137 to 1.077	0.129					
	wx3∼wy2	0.027	0.017 to 0.038	**<0.001 **					
	wy3∼wx2	0.470	−0.137 to 1.077	0.129					
	wx4∼wy3	0.027	0.017 to 0.038	**<0.001 **					
	wy4∼wx3	0.470	−0.137 to 1.077	0.129					
H&Y x UPDRS III	wx2∼wy1	0.008	0.003 to 0.014	**0.002 **	RIx∼∼RIy		1.549	1.076 to 2.022	**<0.001 **
	wy2∼wx1	0.047	−1.638 to 1.731	0.957					
	wx3∼wy2	0.008	0.003 to 0.014	**0.002 **					
	wy3∼wx2	0.047	−1.638 to 1.731	0.957					
	wx4∼wy3	0.008	0.003 to 0.014	**0.002 **					
	wy4∼wx3	0.047	−1.638 to 1.731	0.957					
MOCA x GDS	wx2∼wy1	−0.159	−0.252 to −0.067	**0.001 **	RIx∼∼RIy		−1.082	−1.815 to −0.348	**0.004 **
	wy2∼wx1	−0.117	−0.209 to −0.025	**0.013 **					
	wx3∼wy2	−0.159	−0.252 to −0.067	**0.001 **					
	wy3∼wx2	−0.117	−0.209 to −0.025	**0.013 **					
	wx4∼wy3	−0.159	−0.252 to −0.067	**0.001 **					
	wy4∼wx3	−0.117	−0.209 to −0.025	**0.013 **					
MOCA x STAI	wx2∼wy1	−0.031	−0.046 to −0.017	**<0.001 **	RIx∼∼RIy		−6.410	−10.820 to −2.000	**0.004 **
	wy2∼wx1	−0.709	−1.152 to −0.265	**0.002 **					
	wx3∼wy2	−0.031	−0.046 to −0.017	**<0.001 **					
	wy3∼wx2	−0.709	−1.152 to −0.265	**0.002 **					
	wx4∼wy3	−0.031	−0.046 to −0.017	**<0.001 **					
	wy4∼wx3	−0.709	−1.152 to −0.265	**0.002 **					
MOCA x UPDRS II	wx2∼wy1	−0.134	−0.192 to −0.077	**<0.001 **	RIx∼∼RIy		−2.812	−5.563 to −0.062	**0.045 **
	wy2∼wx1	−0.277	−0.465 to −0.089	**0.004 **					
	wx3∼wy2	−0.134	−0.192 to −0.077	**<0.001 **					
	wy3∼wx2	−0.277	−0.465 to −0.089	**0.004 **					
	wx4∼wy3	−0.134	−0.192 to −0.077	**<0.001 **					
	wy4∼wx3	−0.277	−0.465 to −0.089	**0.004 **					
MOCA x UPDRS III	wx2∼wy1	−0.032	−0.051 to −0.013	**0.001 **	RIx∼∼RIy		−5.412	−9.145 to −1.679	**0.004 **
	wy2∼wx1	−0.489	−0.784 to −0.195	**0.001 **					
	wx3∼wy2	−0.032	−0.051 to −0.013	**0.001 **					
	wy3∼wx2	−0.489	−0.784 to −0.195	**0.001 **					
	wx4∼wy3	−0.032	−0.051 to −0.013	**0.001 **					
	wy4∼wx3	−0.489	−0.784 to −0.195	**0.001 **					
GDS x STAI	wx2∼wy1	0.029	0.010 to 0.048	**0.003 **	RIx∼∼RIy		25.986	20.983 to 30.989	**<0.001 **
	wy2∼wx1	1.029	0.411 to 1.647	**0.001 **					
	wx3∼wy2	0.029	0.010 to 0.048	**0.003 **					
	wy3∼wx2	1.029	0.411 to 1.647	**0.001 **					
	wx4∼wy3	0.029	0.010 to 0.048	**0.003 **					
	wy4∼wx3	1.029	0.411 to 1.647	**0.001 **					
GDS x UPDRS II	wx2∼wy1	0.103	0.043 to 0.162	**0.001 **	RIx∼∼RIy		3.179	1.747 to 4.611	**<0.001 **
	wy2∼wx1	0.261	0.082 to 0.440	**0.004 **					
	wx3∼wy2	0.103	0.043 to 0.162	**0.001 **					
	wy3∼wx2	0.261	0.082 to 0.440	**0.004 **					
	wx4∼wy3	0.103	0.043 to 0.162	**0.001 **					
	wy4∼wx3	0.261	0.082 to 0.440	**0.004 **					
GDS x UPDRS III	wx2∼wy1	0.005	−0.022 to 0.032	0.731	RIx∼∼RIy		0.874	−1.415 to 3.162	0.454
	wy2∼wx1	−0.048	−0.478 to 0.381	0.825					
	wx3∼wy2	0.005	−0.022 to 0.032	0.731					
	wy3∼wx2	−0.048	−0.478 to 0.381	0.825					
	wx4∼wy3	0.005	−0.022 to 0.032	0.731					
	wy4∼wx3	−0.048	−0.478 to 0.381	0.825					
STAI x UPDRS II	wx2∼wy1	0.445	0.180 to 0.709	**0.001 **	RIx∼∼RIy		20.178	12.223 to 28.133	**<0.001 **
	wy2∼wx1	0.010	−0.016 to 0.036	0.456					
	wx3∼wy2	0.445	0.180 to 0.709	**0.001 **					
	wy3∼wx2	0.010	−0.016 to 0.036	0.456					
	wx4∼wy3	0.445	0.180 to 0.709	**0.001 **					
	wy4∼wx3	0.010	−0.016 to 0.036	0.456					
STAI x UPDRS III	wx2∼wy1	−0.014	−0.142 to 0.115	0.836	RIx∼∼RIy		11.490	−2.181 to 25.161	**0.100 **
	wy2∼wx1	−0.045	−0.106 to 0.017	0.155					
	wx3∼wy2	−0.014	−0.142 to 0.115	0.836					
	wy3∼wx2	−0.045	−0.106 to 0.017	0.155					
	wx4∼wy3	−0.014	−0.142 to 0.115	0.836					
	wy4∼wx3	−0.045	−0.106 to 0.017	0.155					
UPDRS II x UPDRS III	wx2∼wy1	0.026	−0.013 to 0.065	0.188	RIx∼∼RIy		9.014	3.885 to 14.143	**0.001 **
	wy2∼wx1	0.377	0.129 to 0.625	**0.003 **					
	wx3∼wy2	0.026	−0.013 to 0.065	0.188					
	wy3∼wx2	0.377	0.129 to 0.625	**0.003 **					
	wx4∼wy3	0.026	−0.013 to 0.065	0.188					
	wy4∼wx3	0.377	0.129 to 0.625	**0.003 **					

*Note*: wx∼wy: Represents a cross‐lagged regression path from the within‐person component of variable Y (wy) to the within‐person component of variable X (wx). The numbers following x and y indicate the measurement wave (e.g., wx2∼wy1 means the within‐person component of X at wave 2 is predicted by the within‐person component of Y at wave 1). RIx∼∼RIy: Represents the covariance between the random intercepts of variable X and variable Y.

Overall, the constrained model showed better results due to either estimation or misspecification problems in the basic model or a better fit of the constrained model. Specifically, the constrained models demonstrated better fit for H&Y x GDS, H&Y x STAI, and GDS x STAI. The Chi‐bar‐squared difference tests confirmed no significant deterioration in fit when equality constraints were imposed, supporting the assumption that the stability and direction of relationships between variables remain consistent over the study period. Given the better fit, the results presented are based on the constrained RI‐CLPMs.

The constrained model results reveal significant relationships across multiple symptom domains, differentiating between time‐invariant, between‐person associations and dynamic, within‐person temporal effects.

### 3.3 Between‐Person, Time‐Invariant Associations (Random Intercept Covariances)

The random intercept covariances (RIx∼∼RIy) capture the stable, “trait‐level” association between variables across individuals over the study period. A significant positive covariance was observed between the random intercepts of depressive symptoms (GDS) and both anxiety (STAI) (cov = 25.986, *p < *0.001) and self‐reported physical function (MDS‐UPDRS Part II) (cov = 3.179, *p<*0.001). This indicates that individuals with a higher average level of depression also tended to have higher average anxiety and more severe self‐reported motor symptoms. A similar positive covariance was found between the Hoehn & Yahr stage and both depressive (cov = 0.146, *p = *0.012) and anxiety (cov = 0.764, *p = *0.024) symptoms, suggesting individuals at a more advanced PD stage experienced greater psychological distress on average. Conversely, significant negative covariances were found between the random intercept for cognition (MoCA) and all other symptom measures: GDS (cov = −1.082, *p = *0.004), STAI (cov = −6.410, *p = *0.004), MDS‐UPDRS Part II (cov = −2.812, *p = *0.045), and MDS‐UPDRS Part III (cov = −5.412, *p = *0.004). These results highlight that individuals who maintained better cognitive function throughout the study tended to have lower overall levels of depression, anxiety, and both self‐reported and clinician‐rated motor symptoms. These time‐invariant findings collectively demonstrate a stable co‐occurrence of symptoms, where a higher chronic burden in one domain across individuals is associated with a higher burden in others.

### 3.4 Within‐Person, Temporal Dynamics (Cross‐Lagged Paths)

The cross‐lagged paths, represented by wx∼wy, reflect the longitudinal, directional influence of fluctuations in one variable on subsequent fluctuations in another, with stable individual differences accounted for. These findings represent the core temporal dynamics of symptom interaction.

A robust, bidirectional cross‐lagged relationship was found between depressive symptoms and self‐reported physical function. Specifically, a change in an individual's GDS score at one time point significantly predicted a subsequent change in their MDS‐UPDRS Part II score (Beta = 0.103, *p = *0.001). Conversely, a change in a person's MDS‐UPDRS Part II score also significantly predicted a subsequent change in their GDS score (Beta = 0.261, *p = *0.004). This pattern indicates a self‐reinforcing “vicious cycle” where worsening depression and worsening self‐reported physical function dynamically influence each other over time.

In contrast, the relationship between anxiety and self‐reported motor symptoms was found to be unidirectional. A significant cross‐lagged path was identified from self‐reported physical function (MDS‐UPDRS Part II) to anxiety (STAI) (Beta = 0.445, *p = *0.001), but no significant reciprocal path was found. This suggests that increases in a person's self‐reported motor symptoms may precede and contribute to an increase in their anxiety levels, but the increase in anxiety does not, in turn, significantly influence subsequent self‐reported motor symptoms in the future.

A notable finding was the temporal relationship between a person's self‐reported physical function and their clinician‐rated motor function. The model revealed a significant unidirectional cross‐lagged path from self‐reported physical function (MDS‐UPDRS Part II) to clinician‐rated motor function (MDS‐UPDRS Part III) (Beta = 0.377, *p = *0.003). There was no significant reciprocal path. This suggests that changes in an individual's perception of their functional abilities may precede and predict a subsequent, more objectively measurable decline in their motor symptoms.

The relationship between cognition (MoCA) and all other symptom domains was consistently bidirectional. For example, a change in MoCA scores significantly predicted a subsequent change in both depressive symptoms (Beta = −0.159, *p = *0.001) and anxiety (Beta = −0.031, *p<*0.001), while a change in these psychological symptoms also significantly predicted a subsequent change in MoCA scores. Similar reciprocal relationships were found between cognition and both self‐reported (MDS‐UPDRS Part II) and clinician‐rated (MDS‐UPDRS Part III) motor function.

Finally, no significant cross‐lagged paths were found between global disease stage (H&Y) and either depressive or anxiety symptoms, or between these psychological symptoms and clinician‐rated motor function. This indicates that while there is a stable, average association between these variables (as shown by the random intercept covariances), changes in psychological symptoms do not dynamically precede or predict changes in the objective, observable signs of the disease as rated by a clinician.

## 4 Discussion

This analysis provides the first longitudinal evidence of a dynamic, bidirectional relationship between depressive symptoms and a person's subjective experience of their physical function in PD. Previous cross‐sectional studies had established a correlation between these factors, but the temporal dynamics remained unclear (Hodgson et al. 2025a). The observed reciprocal cross‐lagged paths indicate a clinically significant feedback loop, often referred to as a “vicious cycle,” where a worsening of depression actively contributes to a decline in perceived physical function, and that subsequent functional decline, in turn, reinforces and exacerbates the experience of a depressive state.

The mechanisms driving this cycle are likely complex. A person experiencing a decline in mood may lose motivation for physical activity and social engagement, leading to a real and perceived reduction in their ability to perform daily tasks (Fried and Nesse 2015). This can also be compounded by decreased adherence to medication regimens or rehabilitation therapies (DiMatteo et al. 2000), further limiting functional capacity. As the person's functional abilities decrease, it can lead to feelings of frustration, loss of independence, and hopelessness, which are core components of depression (Moussavi et al. 2007). This perpetuating cycle highlights that depressive symptoms are not merely a passive consequence of physical decline but are proactive and influential drivers of the lived experience of disability. This finding demonstrates the necessity of addressing both psychological and physical health concurrently to break this negative feedback loop.

A particularly powerful finding of this study is the apparent dissociation between the influence of psychological factors on an individual's lived experience of their condition in comparison to the objective, observable motor signs of the disease. While a strong bidirectional relationship was found between depressive symptoms and self‐reported physical function, no similar temporal or directional relationship was observed between psychological symptoms and clinician‐rated motor function. This indicates that changes in a person's mood do not dynamically precede or predict changes in the underlying neurodegenerative pathology that presents as objective motor signs like tremor or rigidity. Instead, psychological symptoms appear to be a primary determinant of how a person perceives and reports their physical abilities and the overall impact of the disease on their daily life.

This distinction validates the importance of PROs as a unique and non‐redundant measure of disease impact. Patient self‐assessment captures a dimension of the disease experience that is distinct from a clinician's objective examination (Mokkink et al. 2010). This separation reveals a novel and clinically impactful dynamic in which an individual's self‐awareness of their motor experiences can serve as a leading indicator of subsequent objective decline. The unidirectional cross‐lagged path from self‐reported disability to clinician‐rated motor severity suggests that a patient's perception of subtle functional changes precedes a later, more objectively measurable decline. This changes the paradigm for how PROs are viewed in PD care, elevating their status from descriptive tools to potentially predictive and prognostic instruments that can indicate future functional decline earlier than objective measures.

This analysis also highlights the role of cognition as a dynamic marker in the progression of PD symptoms. The models examining the relationship between cognition and all other symptom domains revealed significant and consistently bidirectional relationships. For example, cognitive decline can impair a person's ability to cope with their physical and emotional challenges (Mukku et al. 2021), leading to a further worsening of both mood and motor symptoms (Ziemssen and Reichmann 2007). Conversely, a person experiencing more severe physical and emotional challenges may face a greater cognitive burden (Verbaan et al. 2007), which can further impair their cognitive performance (Starkstein et al. 1992). This tight interdependency suggests a complex, multidirectional feedback loop where cognition is not just another symptom but a core moderator of the entire disease experience. This contrasts with the more static, unidirectional influence of worsening motor symptoms on the progression of global disease stage (via H&Y), and the lack of a dynamic link between H&Y stage and mood symptoms, which further highlights the role of cognitive function in the lived experience of PD progression.

### 4.1 Clinical Implications and Recommendations

The findings from this study have significant clinical implications for the management and therapeutic support for people living with PD. The presence of robust, bidirectional feedback loops between physical function, cognition, and mental health provides a strong evidence base for integrated, patient‐centered care models that address both motor and non‐motor symptoms concurrently. Given the powerful and bidirectional link between depressive symptoms and functional decline, regular and broad screening for psychological symptoms and cognitive decline should be a standard component of PD care. Early identification through routine screening could allow for timely psychotherapeutic or pharmacological intervention, which may not only improve mood but also help disrupt the negative feedback loop and potentially slow the rate of self‐reported functional decline. Physiotherapy assessment of anxiety and depression has been suggested in various conditions, such as rheumatoid arthritis and Fibromyalgia (Vancampfort et al. 2025d; Vancampfort et al. 2025a), but may also be relevant for physiotherapy practice in PD. It has been proposed that physiotherapists have an important role to play in closing the mind‐body divide and that the PHQ‐2 and GAD‐2 offer a pragmatic approach to screening for anxiety and depression in routine physiotherapy practice (Stubbs et al. 2025; Vancampfort et al. 2025b). Evidence suggests that physiotherapists do not currently assess psychological symptoms as part of their routine practice when working with individuals living with PD (Hodgson et al. 2025c; Hodgson et al. 2025b).

Furthermore, the predictive value of PROs, such as the MDS‐UPDRS Part II, should be incorporated into routine clinical practice. These measures offer valuable data that can predict future functional decline earlier than objective measures, guiding the timing of more intensive interventions or a re‐evaluation of the treatment plan. Furthermore, our results suggest a predictive value of self‐reported measures of physical function in future symptoms of anxiety and depression, which could act as a trigger to recommend future psychological interventions. The findings highlight that an effective PD management strategy must extend beyond the clinic and incorporate the patient's lived experience as a vital source of data.

The reciprocal relationship between physical and psychological health in PD, as highlighted by our findings, carries significant implications for clinical training across disciplines. For clinicians, including physiotherapists, there is a need to integrate a more holistic, biopsychosocial approach into educational programs. This is further justified by the stable co‐occurrence of symptoms (random intercept covariances), which shows that a higher chronic burden in one domain (e.g., motor symptoms/PD stage) is consistently linked to a higher burden in others (e.g., depression/anxiety). This reinforces the need for physiotherapists to move beyond a sole focus on motor impairment and explicitly incorporate mental health and the social determinants of health into their care (Vancampfort et al. 2025c). Current literature suggests a notable gap in physiotherapy education, where practitioners and students often report a lack of confidence and training in assessing and managing mental health symptoms despite acknowledging their importance (Furness et al. 2025; Alotaibi et al. 2024). This may hinder their ability to provide comprehensive, patient‐centered care and may contribute to poorer outcomes for individuals with co‐occurring physical and mental health conditions. Therefore, it is essential for training to emphasize not only the physical aspects of clinical presentations, but also to provide robust education in psychological assessment, communication skills, and collaborative care models.

### 4.2 Limitations and Future Research Directions

Despite the valuable insights provided, this study is not without limitations. The use of a secondary dataset, while providing a large cohort, meant that it was not possible to control for all potential confounding variables or the specific timing and nature of interventions. The amount of missing data, particularly at later timepoints, could impact the power of the analysis, although a robust estimator was used to mitigate this issue. Additionally, the PPMI cohort, while large, may not be fully representative of the broader PD population, as participants in such studies are often highly motivated and monitored individuals.

Future research should investigate the specific underlying mechanisms of the observed bidirectional paths, perhaps through more frequent assessments to capture the micro‐longitudinal dynamics of symptom change. It would also be valuable to explore the impact of specific interventions on the stability and direction of these relationships. Models used in future research should consider including time‐dependent covariates that would vary over the timespan of the study to provide more detailed insights into potential symptom interactions. Lastly, future studies should consider the use of other objective measures of physical function beyond the MDS‐UPDRS Part III, as the PPMI dataset does not necessarily represent the outcomes most used in clinical practice.

## 5 Conclusion

Our study used RI‐CLPM to clarify the longitudinal and directional relationships between physical function and psychological symptoms in PD. The findings reveal that depression is strongly and bidirectionally linked to an individual's self‐reported functional decline, but not to objective, clinician‐rated motor signs of the disease. This suggests that a person's psychological state and their perceived physical abilities are locked in a “vicious cycle” where each amplifies the other. We also found that poorer self‐reported motor experiences can act as an indicator of subsequent objective motor deterioration, while also predicting subsequent increases in both anxiety and depression. This work demonstrates the high importance of a holistic, integrated approach to care that not only addresses objective motor signs but also proactively screens for and acts upon patient‐reported outcomes.

## Author Contributions

**Philip Hodgson**: conceptualization, data curation, formal analysis, investigation, methodology, project administration, resources, writing – original draft, writing – review and editing, software, validation, visualization; **Hanna Glandorf**: conceptualization, data curation, formal analysis, methodology, resources, writing – review and editing, software, validation; **Adam Hodgson**: visualization, writing – review and editing, software, validation; **Charikleia Sinani**: resources, writing – review and editing, supervision; **Alastair Jordan**: resources, writing – review and editing, supervision; **Divine Charura**: resources, writing – review and editing, supervision.

## Funding

The authors have nothing to report.

## Ethics Statement

Ethical approval was obtained via York St John University, School of Science, Technology, and Health, prior to undertaking data analysis (Reference: ETH2324‐0171). Informed patient consent was not necessary for this work. We confirm that we have read the Journal's position on issues involved in ethical publication and affirm that this work is consistent with those guidelines.

## Conflicts of Interest

The authors declare that there are no conflicts of interest relevant to this work.

## Supporting information



Supplementary File 1: Correlation Coefficients Matrix

Supplementary File 2: Filterable Correlation Coefficients Table

Supplementary Table 1: Fit Indices for RI‐CLPM

Supplementary Table 2: Comparison of basic and constrained models

## Data Availability

The data that support the findings of this study are available from the Parkinson's Progression Markers Initiative (PPMI). Restrictions apply to the availability of these data, which were used under license for this study. Data are available at https://www.ppmi‐info.org/access‐data‐specimens/download‐data with the permission of PPMI.
