# Supplementary material for: Longitudinal Dynamics of Physical Function With Anxiety and Depression in Parkinson's Disease: A Cross‐Lagged Panel Analysis of the PPMI Dataset
Source: Brain Behav. 2026 Feb 12;16(2):e71257. doi: 10.1002/brb3.71257 (PMC12895980; doi:10.1002/brb3.71257)
Supplement: Supplementary file 3 — Supplementary Table 1: Fit Indices for RI‐CLPM [file BRB3-16-e71257-s002.docx]

Supplementary Table S1: Fit Indices for RI-CLPM

| **Model** *(BL, V04, V06, V08)* | **Model Fit Measures** | | | | | | | | | |
| --- | --- | --- | --- | --- | --- | --- | --- | --- | --- | --- |
|  | **Basic Model** | | | | | **Constrained Model** | | | | |
|  | Chi Square (Scaled) | Robust CFI | Robust TFI | Robust RMSEA | SRMR | Chi Square (Scaled) | Robust CFI | Robust TFI | Robust RMSEA | SRMR |
| H&Y x MOCA | 0.000 | 0.988 | 0.961 | 0.074 | 0.029 | 0.000 | 0.975 | 0.959 | 0.076 | 0.047 |
| H&Y x GDS | 0.003 | 0.990 | 0.970 | 0.063 | 0.025 | 0.012 | 0.988 | 0.981 | 0.050 | 0.039 |
| H&Y x STAI | 0.000 | 0.989 | 0.965 | 0.072 | 0.024 | 0.001 | 0.987 | 0.979 | 0.055 | 0.038 |
| H&Y x UPDRS II | Model Estimation Failed | | | | | 0.016 | 0.990 | 0.984 | 0.051 | 0.047 |
| H&Y x UPDRS III | 0.001 | 0.989 | 0.965 | 0.046 | 0.027 | 0.000 | 0.983 | 0.972 | 0.069 | 0.050 |
| MOCA x GDS | 0.028 | 0.996 | 0.987 | 0.048 | 0.024 | 0.000 | 0.986 | 0.977 | 0.063 | 0.044 |
| MOCA x STAI | 0.020 | 0.997 | 0.989 | 0.034 | 0.024 | 0.000 | 0.984 | 0.973 | 0.071 | 0.042 |
| MOCA x UPDRS II | 0.013 | 0.994 | 0.981 | 0.060 | 0.025 | 0.000 | 0.981 | 0.968 | 0.078 | 0.046 |
| MOCA x UPDRS III | 0.000 | 0.986 | 0.957 | 0.070 | 0.024 | 0.000 | 0.971 | 0.952 | 0.091 | 0.047 |
| GDS x STAI | 0.083 | 0.999 | 0.995 | 0.033 | 0.023 | 0.300 | 0.999 | 0.999 | 0.014 | 0.031 |
| GDS x UPDRS II | 0.049 | 0.997 | 0.989 | 0.044 | 0.020 | 0.001 | 0.988 | 0.981 | 0.060 | 0.037 |
| GDS x UPDRS III | 0.000 | 0.990 | 0.970 | 0.058 | 0.022 | 0.000 | 0.982 | 0.971 | 0.068 | 0.040 |
| STAI x UPDRS II | Model Estimation Failed | | | | | 0.000 | 0.989 | 0.982 | 0.059 | 0.035 |
| STAI x UPDRS III | 0.011 | 0.995 | 0.985 | 0.036 | 0.019 | 0.000 | 0.988 | 0.980 | 0.058 | 0.037 |
| UPDRS II x UPDRS III | 0.000 | 0.991 | 0.973 | 0.072 | 0.024 | 0.000 | 0.984 | 0.973 | 0.072 | 0.049 |
