# Supplementary material for: Longitudinal Dynamics of Physical Function With Anxiety and Depression in Parkinson's Disease: A Cross‐Lagged Panel Analysis of the PPMI Dataset
Source: Brain Behav. 2026 Feb 12;16(2):e71257. doi: 10.1002/brb3.71257 (PMC12895980; doi:10.1002/brb3.71257)
Supplement: Supplementary file 4 — Supplementary Table 2: Comparison of basic and constrained models [file BRB3-16-e71257-s003.docx]

Table 5: Comparison of basic and constrained models

|  | **Chi-Bar-Squared Value** | **p-value** | **Outcome** |
| --- | --- | --- | --- |
| H&Y x MOCA | Indications of model misspecification in the basic model | | n/a |
| H&Y x GDS | 16.345 | 0.615 | Constraint model fits better |
| H&Y x STAI | 5.519 | 0.998 | Constraint model fits better |
| H&Y x UPDRS II | Indications of model misspecification in the basic model | | n/a |
| H&Y x UPDRS III | Indications of model misspecification in the basic model | | n/a |
| MOCA x GDS | Indications of model misspecification in the basic model | | n/a |
| MOCA x STAI | Indications of model misspecification in the basic model | | n/a |
| MOCA x UPDRS II | Indications of model misspecification in the basic model | | n/a |
| MOCA x UPDRS III | Indications of model misspecification in the basic model | | n/a |
| GDS x STAI | 11.007 | 0.899 | Constraint model fits better |
| GDS x UPDRS II | Indications of model misspecification in the basic model | | n/a |
| GDS x UPDRS III | Indications of model misspecification in the basic model | | n/a |
| STAI x UPDRS II | Indications of model misspecification in the basic model | | n/a |
| STAI x UPDRS III | Indications of model misspecification in the basic model | | n/a |
| UPDRS II x UPDRS III | Indications of model misspecification in the basic model | | n/a |
